# Prostate Cancer Radiation Therapy: What Do Clinicians Have to Know?

**DOI:** 10.1155/2016/6829875

**Published:** 2016-12-28

**Authors:** Ben G. L. Vanneste, Evert J. Van Limbergen, Emile N. van Lin, Joep G. H. van Roermund, Philippe Lambin

**Affiliations:** ^1^Department of Radiation Oncology (MAASTRO Clinic), GROW School for Oncology and Developmental Biology, Maastricht University Medical Centre, Maastricht, Netherlands; ^2^Department of Urology, Maastricht University Medical Centre, Maastricht, Netherlands

## Abstract

Radiotherapy (RT) for prostate cancer (PC) has steadily evolved over the last decades, with improving biochemical disease-free survival. Recently population based research also revealed an association between overall survival and doses ≥ 75.6 Gray (Gy) in men with intermediate- and high-risk PC. Examples of improved RT techniques are image-guided RT, intensity-modulated RT, volumetric modulated arc therapy, and stereotactic ablative body RT, which could facilitate further dose escalation. Brachytherapy is an internal form of RT that also developed substantially. New devices such as rectum spacers and balloons have been developed to spare rectal structures. Newer techniques like protons and carbon ions have the intrinsic characteristics maximising the dose on the tumour while minimising the effect on the surrounding healthy tissue, but clinical data are needed for confirmation in randomised phase III trials. Furthermore, it provides an overview of an important discussion issue in PC treatment between urologists and radiation oncologists: the comparison between radical prostatectomy and RT. Current literature reveals that all possible treatment modalities have the same cure rate, but a different toxicity pattern. We recommend proposing the possible different treatment modalities with their own advantages and side-effects to the individual patient. Clinicians and patients should make treatment decisions together (*shared decision-making*) while using patient decision aids.

## 1. Introduction

Prostate cancer (PC) is the most common cancer among males in the Western world, with more than 1.11 million new cases diagnosed in 2012 and 307,000 deaths [1, 2]. The lifetime risk of developing PC is 1 in 8 [3]. It is expected that the incidence will substantially increase in the coming decades due to the aging population, which makes it a huge health care problem. The total economic costs of PC in Europe are estimated to exceed €8.43 billion [4]. One of the biggest challenges in the 21st century will be to offer the best individualised treatment at reasonable costs.

External-beam radiotherapy (EBRT) and brachytherapy (BT) are potentially curative therapies for PC. RT has undergone tremendous improvements in the last decades. Dose escalation in prostate EBRT leads to improved locoregional control, biochemical disease-free survival (bDFS), distant metastasis-free survival, PC specific mortality, and even overall survival in intermediate- and high-risk PC [5–11]. However, dose escalation is limited by toxicity of surrounding healthy tissues, and therefore improved tumour control is expected to come at the cost of higher toxicity, greatly impacting patients' quality of life [12–14]. However, dose escalation is possible due to advances in different RT techniques, sophisticated computer-based treatment planning, and/or development of extra devices, avoiding increased dose delivery to the surrounding healthy tissue. The purpose of this article is to provide insight into the enormous improvements in RT techniques to practicing clinicians and primary care doctors and to develop a greater comfort level when referring patients to a radiation oncologist. Furthermore, it provides an overview of an important discussion issue concerning RT from a clinician's perspective: the comparison between operation and RT.

## 2. Overview of External Beam Radiation Treatments

In EBRT a dose of ionising radiation is generated by an external X-ray source. In the past this was a cobalt-60 source machine, but nowadays a high-tech tele-therapy unit is used for this purpose [15, 16]. Linear accelerators are the source of electronic induced irradiation. The radiation beam leaves the linear accelerator by a gantry. Different options of machines are commercially available: a traditional linear accelerator where the gantry can rotate around the patient (Arc therapy). Other possibilities are tomotherapy (=helical therapy) where the radiation dose is delivered slice-by-slice [17], or cyberknife (=a robotic radiosurgery system) where the location of the prostate is identified during treatment and active corrections are made for movements of the prostate during treatment delivery [18]. Evolving radiation techniques as protons and carbon ions are also introduced and are discussed below. Over the last 20 years the methods of delivering a dose of ionising radiation to a target area have changed incrementally.

**Figure 1:**
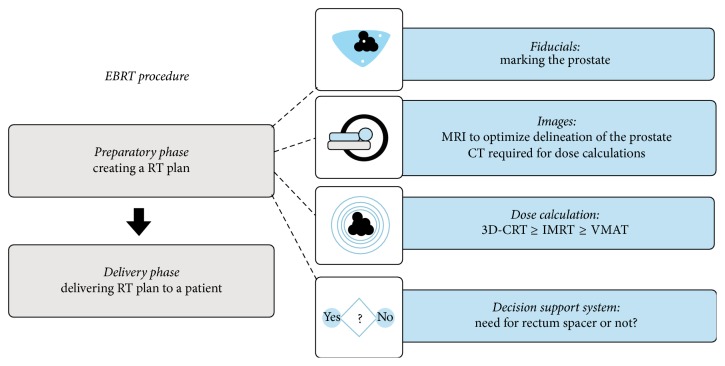
Overview of an EBRT procedure.

An EBRT procedure consists of 2 main parts (Figure 1).

First, in a preparatory phase an RT plan needs to be created. This process is referred to as RT planning. Secondly, the linear accelerator requires delivering this plan to a patient in an appropriate fashion: the RT dose delivery.

In the preparatory phase, images of the patient are acquired. On these scans the clinical target area is delineated to which the radiotherapy dose is prescribed. In the 90s this area was delineated on conventional planar 2D X-rays, on which the target area (the prostate and seminal vesicles) could only be assumed. Later, CT based planning was introduced [19]. On the latter the target areas are visualised and can be delineated directly leading to up to one-third less geographical miss of the target [20]. Another advantage of CT based planning was that also critical structures like rectal wall and bladder around the target could be visualised and subsequently spared from radiation, by avoiding the X-ray beams to pass through them. Currently an MRI is being integrated more broadly into the planning process. MRI allows us to delineate the prostate more precisely from the pelvic diaphragm, and the base of the prostate can be differentiated more precisely from the seminal vesicles [21, 22]. An additional MRI changes the delineation of the clinical target volume in 18% to 20% of cases compared to CT based planning [23, 24]. Moreover, tumour extension in and outside of the prostate and invasion in the seminal vesicles are better visible on MRI and therefore more often included in the target volumes [24, 25]. Chang and colleagues reported significant volume changes with MRI delineation: extracapsular extension was significantly more incorporated into target volumes with the addition of MRI (40%) in comparison with CT (32%). The seminal vesicles are also more often included: 18% versus 3%, respectively. In addition, CT scans overestimate prostate volume by 10% to 45% [21, 22, 26–32]. Furthermore, an MRI revealed an important decrease of the interobserver delineation variation, especially at the prostatic apex [33]. We expect that a correct delineation of the target volume will result in better treatment outcome, with less toxicity, but until now this is not proven yet.

In addition to improved radiotherapy planning, developments were introduced to verify correct dose delivery during the whole course of RT over the several fractions delivered according to the radiotherapy plan. In earlier times patients were positioned on a linear accelerator using surrogate reference points: external reference points like skin lines or tattoo points or using bony landmarks visualised by conventional plain X-ray photographs taken on the linear accelerator. However, as it is known that the prostate and the seminal vesicles can move independently from these reference points this can be problematic because it could lead to off-target dose delivery, which in turn compromises tumour cure [34, 35]. In earlier times this problem was compensated by expanding the margins of the RT field to minimise the chance of a geographical miss. The downside of this approach was however that this approach leads to a higher volume of irradiation to the surrounding healthy tissues and critical structures. More recently, this problem is tackled by the placement of fiducials (markers) into the prostate before the RT treatment [36–38]. In this way the movement of the prostate can be monitored during treatment, and field setups can be adjusted in case of movement of the prostate ensuring correct dose delivery, even with small safety margins. A comparable methodology is implantation of electromagnetic transponders (Calypso®) [39]. Other image guidance strategies are used but are focused on visualisation of the prostate itself instead of a surrogate (marker): cone-beam computed tomography [40], MRI [41], and ultrasound imaging [42]. The most popular strategy is the use of fiducials because of the easy and quick performance. Disadvantages of the image guidance strategy directly focused on the organ are poor image quality (cone-beam computed tomography, ultrasound) and high costs (MRI). All this leads to the development of dose volume constraints to diminish the chance on rectal and urinary toxicity [13, 43].

As delineation became more accurate and precise, consequently the necessity emerged for better shaping the dose around the target and avoiding the critical structures. In earlier techniques, like 3D-conformal RT, beams were shaped around the tumour contours with a collimator blocking gamma rays out of unwanted areas (i.e., healthy organs). The tumour was irradiated mostly using 4 fields opposed to each other (anteroposterior and lateral opposing fields). The result was a high-dose “box” in the overlap zone of the four bundles. Later, intensity-modulated radiotherapy (IMRT) techniques were introduced. Here the tumour was approached from additional angles, using mobile computer-controlled collimators, creating additional degrees of freedom to shape the high-dose region around the target.

**Figure 2:**
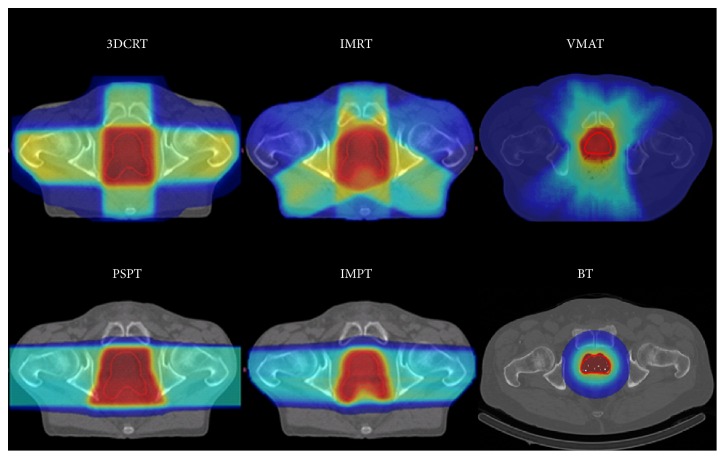
Examples of dose distribution of a 3DCRT, IMRT-5, VMAT, PSPT, IMPT, and a BT treatment plan calculated on the same patient. The red surface represents the high-dose regions, the yellow surface the intermediate-high-dose regions, the dark blue surface the low-dose regions, and the azure blue surface the intermediate-dose regions. 3D-CRT: 3-dimensional conformal radiotherapy; IMRT: intensity modulated radiotherapy; VMAT: volumetric modulated arc therapy; PSPT: passively scattered proton therapy; IMPT: intensity modulated proton therapy; BT: brachytherapy.

Volumetric modulated arc therapy (VMAT) or rapid arc therapy is a relatively novel radiation technique. It is an advanced form of IMRT that delivers a 3D-dose distribution with a 360-degree rotation of the gantry in a single or multiarc treatment. This results in an improved target volume coverage and sparing of normal tissues compared with less modern techniques (Figure 2). VMAT has the advantage of favourable dose distributions. Furthermore, it reduced the monitor units required compared with IMRT and reduced treatment delivery time [44, 45].

These improvements in delineation and more conformal RT technique but also treatment delivery verifications allowed for further dose escalation resulting in higher cure rates with similar or slightly higher toxicity [8, 46–53]. Standard RT uses a daily dose of 1.8 to 2.0 Gy for 39–45 fractions. The updated published randomised phase III trials of dose escalation are summarised in Table 1. The dose escalations revealed a 10 to 20% increase of bDFS. This advantage, however, did not translate into an improvement of overall survival. Besides, Kalbasi and colleagues demonstrated in a huge cohort of patients (42,481) of the National Cancer Data base that dose escalation up to ≥75.6 Gy is associated with improved overall survival in men with intermediate- and high-risk prostate cancer [11].

### 2.1. Hypofractionation

A total dose cannot be delivered in one fraction, since this would produce serious adverse reactions. Therefore, the total dose needs to be split into fractions. Healthy cells can recover themselves from the RT during the interfraction periods, whereas tumour cells are damaged. Hypofractionated (HF) EBRT means a larger dose per fraction with less fractionations, mainly given over a shorter time period, with a lower total dose. This lower total dose has a comparable effect with a higher standard dose in fractions of 2 Gy [54]. The damage is greater in larger fractionations and the total dose is lower for the same effect. To easily compare the different RT schemas all RT schedules are recalculated in standard 2 Gy fractions. Several tools are available to calculate different RT schedules with each other, for example, http://rotoolbox.com/calculators/eqd2/.

HF for PC is traditionally performed in 19 to 28 fractions of 2.5 Gy to 3.4 Gy per fraction. HF has earned increasing attention as it has a higher therapeutic ratio (=the difference between treatment benefits and morbidity) than standard fractionated IMRT, which may theoretically lead to greater local cancer control [55, 56]. Furthermore, HF EBRT ameliorates logistical inconveniences for both patients and their providers. It is particularly useful for patients who benefit logistically from a shortened HF course like patients living at long distance from an RT centre or who have a poor support system [57, 58]. The results of three recently published phase III trials are summarised in Table 2 [59–61]. These trials revealed that HF is well tolerated, albeit with a slight increase in toxicity rates when compared to conventional schedules. No improvement on bDFS has been noticed; however, the follow-up period is possibly insufficient. Further evaluations and reports are expected in the coming years.

### 2.2. Stereotactic Body Radiotherapy

Stereotactic body radiotherapy (SBRT) is an extreme form of HF. Stereotaxy refers to a precise method of target localisation using three-dimensional coordinates derived from medical imaging. SBRT for PC is traditionally performed in 3–7 fractions of 6 Gy to 10 Gy per fraction. SBRT is delivered with even higher than standard precision procedures, for example, a customised body pillow formed by vacuum suction [62]. Just like in conventional EBRT there is an evolution with more dose guidance and higher precision (see above). The available literature consists mainly of several nonrandomised phase II trials. Recently, a large multi-institutional trial of 1100 patients was reported. Separate prospective phase 2 protocols of localised PC patients from different institutes treated between 2003 and 2011 were pooled for analysis [63]. With a median follow-up of 36 months, the five-year bDFS rate was 93%. As this series mostly consisted of low- and intermediate-risk patients and follow-up is still limited, this treatment is only recommended for selected low- and intermediate-risk patients with localised PC. That the* acute *urogenital toxicity seemed higher than conventional EBRT [64] might pose a disadvantage. On the other hand, low* late* urinary and rectal toxicities after median follow-up of three years were reported [65]. Data from published prostate SBRT trials have shown late grade 3 GI and GU toxicities within the 3%. However, this data is preliminary and prospective randomised phase III trials and additional follow-up are required to further clarify the relative differences between both treatment modalities.

## 3. Brachytherapy

BT is an internal RT, where radiation comes from an implanted source, such as seeds or capsules. BT permits an extreme dose escalation far exceeding other RT modalities. Furthermore, no extra treatment margin is necessary for set-up errors. In general, two types of BT are clinically used: low-dose rate (LDR) and high-dose rate (HDR). In LDR radioactive sources are permanently implanted in the prostate, whereas at HDR temporary needles are placed in the prostate in which a radioactive source irradiates the prostate temporarily. Both modalities can be used either as a monotherapy or as a boost with EBRT. Monotherapies are generally used for low- and intermediate-risk PC, whereas combined therapy usually is used for intermediate- and high-risk PC [66]. The logistics are the main advantage of LDR: you can implant it with small shields, whereas HDR is applied in a specialised shielded room for radioprotection issue. LDR has the disadvantage that some extensions are difficult to cover, for example, seminal vesicle extension and extra capsular extension, which can be adequately covered by HDR.

### 3.1. Low-Dose Rate

Permanent seed implantation involves injecting approximately 50–125 radioactive seeds into the prostate depending on the volume [67]. General or spinal anaesthesia is required. The seed implantation is performed under TRUS guidance via the transperineal approach, with the patient placed in dorsal lithotomy position. LDR is accomplished in an outpatient single visit setting. Individual (loose) seeds or stranded seeds (seeds linked together in dissolvable suture material) are used in LDR [66]. Stranded seeds minimise seed migration and improve dose delivery [68, 69]. The planned RT dose is emitted over several months with an average dose rate of 0.1 Gy/h, depending on the specific isotope [70]. Iodine-125 (I-125) and palladium-103 (Pd-103) are mostly used. Pd-103 has a higher dose rate and is more frequently used in the United States. The prescription dose varies from 145 Gy for I-125 to 120 Gy for Pd-103. The BT alone is an option for patients with low- and intermediate-risk disease when there are only limited features, such as a serum PSA between 10 and 20 ng/mL or small volume Gleason score 7 [68, 70].

Grimm et al. conducted a comprehensive literature review to identify over 18,000 papers involving treatment of localised PC published during 2000–2010 [71]. Selection criteria were made based on the following criteria: median follow-up of at least five years (which is still short for PC); patient stratification into pretreatment risk; both clinical and pathological staging; accepted standard definitions for PSA failure; minimum patients number for each risk group which was accepted as 100 for low- and intermediate- and 50 for high-risk group; and results published in peer-review journals only. All the study outcomes were calculated for each risk group and suggested that BT alone, particularly seed implant, provides superior bDFS in low-risk patients. For the intermediate-risk group, combination RT (EBRT + BT) seems to be equal to BT alone. For high-risk patients combination RT with or without androgen deprivation therapy seems to be superior. Furthermore, in a recently reported randomised trial (ASCENDE-RT, NCT00175396), a LDR boost was demonstrated to be much more effective than an EBRT boost in high-risk prostate cancer patients: a 9-year BRFS of 83% versus 63% [72]. However, these results should be interpreted with some caution because this is only published in an abstract form: no mention of image guidance or quality assurance is made, yet. Toxicity rates are also not clearly mentioned in this abstract. Although these results encourage choosing BT as an element of management, it should be remembered that selection bias may play a main role.

### 3.2. High-Dose Rate

With HDR BT, transperineal catheters are first inserted in the prostate under general or spinal anaesthesia. The hollow catheters are connected to an HDR “afterloader” with an isotope, mostly iridium-192 (Ir-192). The dose rate is at least 12 Gy/h. The afterloader machine loads the hollow catheters while the BT team is outside the shielded room for radioprotection issues. This machine pushes a wire connected to the radioactive source into each of the different catheters, one by one under computer-control, utilising stop positions and dwell times according to the plan. After treatment, the afterloader withdraws the sources. After the BT treatment the catheters are removed. No radioactive seeds are left in the body.

HDR is often used in a combination therapy with EBRT. Outcomes are superior to those achieved with EBRT alone [73–77]. One phase III trial is reported by Mount Vernon Hospital where they compared EBRT (55 Gy, 20x) with EBRT (37.5 Gy, 13x) and HDR boost (17 Gy, 2x) [73]. Hoskin et al. demonstrated a 7-year BRFS rate of 75% compared with 61%, respectively, with similar incidence of severe late urinary and rectal morbidity. An ongoing randomised trial (PROBACH, NTR3897) will further evaluate the value of HDR as a boost therapy in intermediate- and high-risk PC.

Another older phase III trial is reported by Sathya and colleagues [78]. They proved that the combination of HDR plus EBRT was superior to EBRT alone for a 5 years BRFS of 71% compared with 39%. This is logic when comparing the total dose schedules to the prostate: the combination therapy was superior with 75 to 80 Gy (comparable with nowadays EBRT schedules) in comparison with EBRT only where the given dose was inferior with 66 Gy and with 2 cm safety margins.

Although the interest in monotherapy HDR is growing, no phase III trials are conducted. Several nonrandomised series are reported on the results of monotherapy HDR in multiple and in single fractions, which are promising.

## 4. New Techniques: Proton Therapy, Carbon Ion

**Figure 3:**
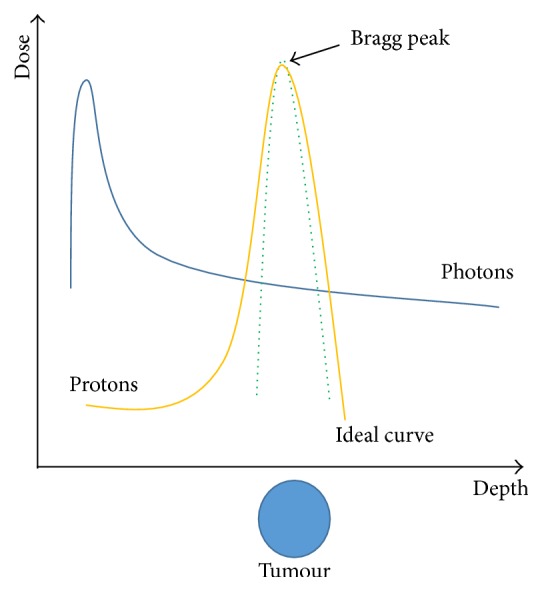
The Bragg peak demonstrating the plots energy loss of ionising radiation during its travel through the body. Maximum energy deposition at the target area (tumour) without energy loss after the target (healthy organs).

Newer RT techniques which utilise heavy particles such as protons and carbon ions have a potential dosimetric benefit of the so-called “Bragg” peak (Figure 3). This means that the maximum dose delivery occurs immediately before the particles come to rest. This means that the maximum effect on the tumour can be determined while minimising the impact on the surrounding healthy tissue. These approaches are currently in development [79–81].

Zietman et al. published the only randomised series currently available, comparing a high- to a low-proton boost, resulting in a significant increase in bDFS in the high-dose arm [8].

Carbon ions seem more efficient than protons which can be explained by the fact that carbon ion beams are twice to three times more effective than protons or photons [82, 83]. Habl and colleagues published an HF schedule using either carbon ions or protons resulting in comparable acute toxicities [84]. Long-term outcome data on these treatments are not yet available. However, until now, no evidence is shown to support the use of protons in preference to conventional RT for patients with prostate cancer; neither technique had been shown to give improved results over the others with respect to disease control or toxicity [85].

An ongoing multi-institutional phase III-randomised trial (PARTIQoL, NCT01617161) evaluates the value of protons in low- and intermediate-risk PC in comparison with IMRT. This trial will probably shed light on the additional value of protons in comparison with conventional IMRT for PC. In any event, we believe the future lies in multifactorial decision support systems calculating for each individual patient the outcome and the cost-effectiveness of the various treatments [86, 87].

## 5. New Devices: Balloon/Spacer

Another way to reduce toxicity is to physically create some space between the healthy organ (rectum) and the targeted area (prostate). As ionising radiation decreases by the inverse square law, even a few millimetres of increased separation can lead to sparing the healthy organ for high doses of radiation.

To spare rectal structures several spacer devices are developed [88]. These can be divided into endorectal balloons and relatively novel rectum spacers. Endorectal balloons are placed into the rectum for each daily treatment. Although the ventral anorectal wall is pushed towards the prostate, the distance from the posterior rectal wall to the prostate is increased with an overall effect proved to be beneficial in RT [89].

**Figure 4:**
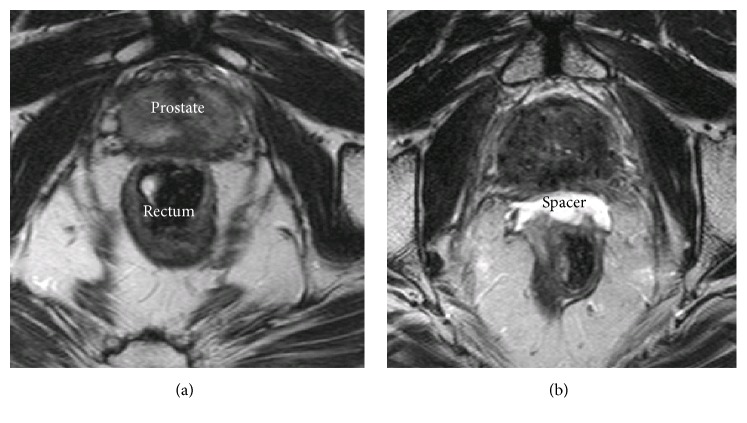
Axial T2-weighted magnetic resonance images of a patient with a hydrogel spacer before injection (a) and after injection (b).

Rectum spacers are implanted as a tissue filler into the anterior perirectal fat to separate the rectum from the prostate (Figure 4). Increasing the prostate-rectum distance displaces the rectal wall away from the prostate and out of the high-dose RT regions. The overall effect is a reduction in the total volume of irradiated rectum and the maximum dose to the rectum. The implantation of such rectum spacers is performed transperineally under real-time TRUS guidance. The insertion procedure can be performed under local, spinal, or general anaesthesia [90]. The implanted rectum spacer remains in place over the course of the RT treatment and the spacer biodegrades naturally within six months after implantation [91]. Different types of rectum spacers have been developed: an absorbable hydrogel, a hyaluronic acid, a collagen, and a saline-filled balloon [91, 92]. Although several studies are available on the acute outcome, dosimetry, and cost-effectiveness of a rectum spacer, the long-term outcomes are not yet clear [93–103]. If the spacer is combined with HF, BT, SBRT, or proton therapy, the reduction of toxicity could be even more expected. Very recently, decision rules based on clinical risk factors solely are identified for which patients a spacer implantation is predicted to be beneficial [104]. However, further research is needed to assess the predictive performance of these decision rules and to generate adequate decision support systems. The available results are encouraging for the design of further clinical trials.

## 6. RT Compared to Surgery

The results of a well-balanced randomised phase III trial comparing RT with RP and active monitoring are very recently reported (PROTECT, NCT02044172) [105, 106]. Hamdy and colleagues compared all those treatments for low-risk localised prostate cancer with a median follow-up of 10 years (1643 patients). Only 17 prostate-cancer-specific mortalities were observed: 8 patients in the active-monitoring group, 5 men in the RP group, and 4 patients in the EBRT group. The differences among the groups were not significant. RP and EBRT were associated with lower incidences of disease progression than active monitoring, respectively, 46 incidences for active therapies compared with 112 man for active-monitoring (*p* < 0.001). Also metastases rates developed more in the active-monitoring group: 33 men in comparison with 13 and 16 for RP and EBRT, respectively (*p* = 0.004). Patient-reported outcomes are also reported: RP had the greatest negative effect on sexual function and urinary continence. EBRT had little effect on urinary continence (urinary voiding and nocturia); however, bowel function was worse. In the active-monitoring group sexual and urinary function declined gradually over years.

All treatments provide an extremely high cure rate. Recently, Lennernäs et al. published the first randomised trial comparing RP with EBRT + HDR [107]. Due to insufficient power and small series (89 patients) no conclusion could be drawn about the efficacy. Nonetheless, some observational data suggest that outcomes with RP lead to better overall and cancer-specific survival than RT [108–112]. Wallis and colleagues recently published a meta-analysis comparing RP with EBRT or BT [108]. They pooled 118,830 patients from 19 studies and concluded that overall and prostate cancer-specific mortality were higher for patients treated with RT compared with RP. Subgroup analyses by risk group, radiation regimen, time period, and follow-up length did not alter the results.

However, all those comparison trials have several limitations. First, patients with greater comorbidity tend to be treated with RT [113]. In addition, comorbidities that have been shown a major impact on survival are not always mentioned [114]. Further, some RT schedules in those trials are using inferior low-dose [115]. Also, a potential bias exists for unaccounted differences between risk groups [116]. Next, baseline characteristics are often different and have a profound impact as differences in the percentage of positive biopsies or Gleason 4 + 3 versus 3 + 4 tumours [116–118]. Furthermore, big meta-analyses are being criticised as the studies synthesised in such analyses do not all pose level 3 evidence [119, 120].

Other data suggest that even either EBRT or BT using adequate dosing schedules and conformal techniques are similar to RP when men with clinically localised PC are stratified based upon clinical tumour stage, pretreatment serum Prostate Specific Antigen, and Gleason score [121, 122]. Kim et al. concluded that outcomes are not inferior to those of RP despite the fact that the EBRT group included more high-risk patients [122]. Grimm et al. conducted a comprehensive literature review to identify all studies involving treatment of localised PC. They even concluded that BT provides superior outcome in patients with low-risk and intermediate-risk disease. High-risk disease revealed the best outcome with combination therapy of EBRT and BT [71]. However, like all comparison trials those have several limitations [123]. First, the endpoint of bDFS is not fair because the definition is different for RP and RT. Further, it is difficult to determine bDFS as a surrogate of cancer-specific survival. Moreover, in the comprehensive literature review of Grimm many RP studies are excluded because they are based on pathology report after RP, which is not possible with RT. Next, many surgical factors can influence oncological outcome and are not reported as innovations in RP (robotic-assisted RP) and caseload volume per institute. Finally, the risk stratification (intermediate-risk group) was more varied amongst articles, thus reflected in significant differences in baseline risk for PSA failure between the treatment methods.

To conclude, one well-controlled randomised phase III trial (PROTECT) randomly assigned men with localised PC to active monitoring, RT, or RP. This trial revealed comparable outcomes for each treatment, but with a different toxicity pattern.

Our belief is that a paradigm shift from current population-based medicine to personalised and participative medicine is underway. This transition is being supported by the development of multifactorial clinical decision support systems based on prediction models of treatment outcome and constantly reevaluated in different patient datasets in order to refine and reoptimise the models, ensuring the continuous utility of the models.

Nowadays, decisions on the most appropriate treatment for each patient are dependent on unique personal patient characteristics and preferences, clinician judgment, and resource availability. Therefore, to achieve the right treatment for each individual, we believe patients and clinicians should make decisions together: shared decision-making (SDM) [124, 125] to embrace truly participative medicine. SDM is an interactive process in which patients and clinicians collaborate in choosing health care, based upon the best available evidence [126–128]. Several studies have reported that patients involved in SDM experience less decisional conflict, improved compliance with treatment, and a greater quality of life with less comorbidities such as anxiety, fatigue, and depression [129]. This has been confirmed in a Cochrane study by Stacey and colleagues [130]. The health care system benefits, also in terms of reduced costs and fewer unnecessary/unwanted procedures [131]. However, the implementation of SDM remains a challenge in health care systems due to numerous barriers [132–134]. These barriers can be divided into patient, clinician, and organisational barriers. Patient barriers include age and attitudes. Older patients tend to prefer a paternalistic model in which treatment decisions are made by the doctor [132]. Of course, a significant part of patients opt for this model while the doctor chooses the ideal treatment for the particular patient. There are also barriers from the health care provider side, such as the perception that SDM is too time-consuming or complicated to pursue [133, 134]. Furthermore, clinicians often unintentionally use jargon. Finally, organisational factors such as a lack of support, time, and resources are also commonly described barriers [133].

**Figure 5:**
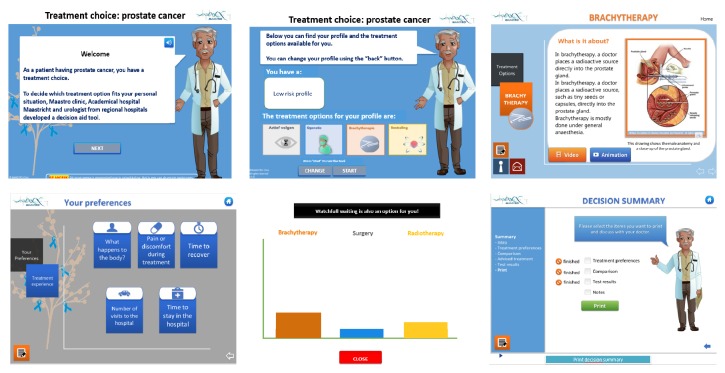
A summary of some screen shots of an interactive PDA for PC (http://www.treatmentchoice.info/). The PDA provides information to the patient of the characteristics of his disease, the available treatments for his own situation, his individual preferences, and a comparison of the possible treatments. It offers a summarised advice based upon the information provided by the patient. The purpose of this is to inform the patient; a final decision is always taken together with the clinician.

Patient decision aids (PDAs) have been developed to overcome these challenges [135]. PDAs supply patients with treatment options, treatment-specific information, and treatment comparison to help patients discover their personal preferences [136] (Figure 5, http://www.treatmentchoice.info/decision-aid-tools.html). PDAs are not developed to promote one option over another or to replace clinician consultation. Instead, they prepare patients to make informed, values-based individual decisions with clinicians (http://ipdas.ohri.ca/) [130, 137].

## 7. Conclusion

During the past 20 years, RT in PC has improved significantly in all areas, including treatment technique, planning, and quality control. Examples of improved RT techniques are image-guided RT, IMRT, VMAT, SBRT, LDR-HDR BT, and protons. Rectum spacers and balloons have been developed to diminish rectal toxicities. Further research is needed to define the value of all these promising new techniques. With those technical implementations the long-term bDFS are improved. We recommend dose escalation up to ≥75.6 Gy (calculated as standard fractionations of 2 Gy). Doses up to 75.6 Gy is associated with improved overall survival in men with intermediate- and high-risk prostate cancer. HF is an attractive therapeutic option, and the randomised phase III trials revealed a slight increase of toxicity rates in comparison to conventional schedules.

An important discussion issue between urologists and radiation oncologists is summarised: the comparison between RP and RT. The results of a well-balanced randomised phase III trial comparing RT with RP and active monitoring are very recently reported. The outcomes of RP and RT are similar, but they differ significantly in terms of the side-effects. We recommend proposing different treatment modalities to the individual patient characteristics and preferences. For each individual, we recommend that clinicians and patients should make decisions together, shared decision-making, while using patient decision aids.

## Figures and Tables

**Table 1:** Updated phase III randomised trials on dose escalation for prostate cancer. All results are statistically significant, except those marked with n.s.

	*N*	Median FU (yrs)	Dose (Gy)	Benefit bDFS (%)	Toxicity GI (%)	Toxicity GU (%)
*MD Anderson*						
Kuban et al. 2008	301	8.7	70 versus 78	59 versus 78	13 versus 26	13 versus 8^n.s.^
*MGH*						
Michalski et al. 2015	1499	7	70.2 versus 79.2	57 versus 74	16 versus 22	10 versus 15
*Dutch trial*						
Heemsbergen et al. 2014	669	9.1	68 versus 78	61 versus 69	25 versus 35	40 versus 41^n.s.^
*Royal Masden*						
Dearnaley et al. 2014	843	10	64 versus 74	43 versus 55	24 versus 33	8 versus 11^n.s.^
*GETUG*						
Beckendorf et al. 2011	306	5.1	70 versus 80	68 versus 76.5	14 versus 19.5	10 versus 17.5

bDFS: biochemical disease-free survival. n.s.: not significant.

**Table 2:** Updated phase III randomised trials on hypofractionation for prostate cancer. All results are statistically significant, except those marked with n.s.

	*N*	Median FU (yrs)	Dose (Gy) per fraction	Benefit bDFS (%)	Toxicity Gr2 GI (%)	Toxicity Gr2 GU (%)
*Dutch trial*						
Aluwini et al. 2015	820	5	39 × 2 versus 19 × 3.4	77 versus 80^n.s.^	Equal; 13	22 versus 23
*RTOG 0415*						
Lee et al. 2016	1092	5.8	41 × 1.8 versus 28 × 2.5	85.3 versus 86.3	11.4 versus 18.3	20.5 versus 26.2
*CHHiP*						
Dearnaley et al. 2016	3163	5.1	37 × 2 versus 20 × 3 versus 19 × 3	88.3 versus 90.6 versus 85.9	Equal; 2^n.s.^	11 versus 13^n.s.^

bDFS: biochemical disease-free survival; CHHiP: conventional or hypofractionated high dose intensity modulated radiotherapy in prostate cancer; n.s.: not significant; Gr2: grade 2 or more toxicity.
